# Metastatic Renal Cell Carcinoma in an Ovarian Benign Mixed Mucinous Cystadenoma and Brenner Tumour: A Case Report

**DOI:** 10.1155/2012/523231

**Published:** 2012-11-27

**Authors:** Amar Ibrahim, Mohammad Salih Al-Jafari

**Affiliations:** Pathology Department, Warrington and Halton Hospitals NHS Foundation Trusts, Lovely Lane, Warrington, Cheshire WA5 1QG, UK

## Abstract

This is a case of a 71-year-old woman, who presented with a large abdominal mass. Microscopic examination revealed metastatic renal cell carcinoma in an otherwise massively enlarged benign ovarian tumour of mixed mucinous cystadenoma and Brenner tumour. Clinicopathological and radiological assessment together with a thorough evaluation of gross and histological features, including the use of immunohistochemical stains, is essential to confirm the diagnosis and rule out the possibility of primary clear cell carcinoma of ovary, as this has important prognostic and therapeutic implications.

## 1. Introduction

Renal cell carcinoma (RCC) is the most common malignant tumour of the kidney. It constitutes 75% of renal neoplasms in adults and 3% of all adult malignancies [1]. Approximately 30% of patients present with distant metastasis at the time of diagnosis [1, 2]. Metastatic renal cell carcinoma to the ovary is uncommon, and so far only small number of cases has been reported in the literature [3]. Furthermore, the presence of metastatic renal cell carcinoma in an existing benign mixed mucinous cystadenoma and Brenner tumor is an extremely rare occurrence. 

## 2. Case Presentation

A 71-year-old woman presented clinically with abdominal distention and weight loss. On physical examination, the patient had a large abdominal mass. Blood biochemistry showed Urea 6.5 mmol/L, Creatinine 77 umol/L, Sodium: 136 mmol/L, Potassium 5.2 mmol/L, and GFR 79 mL/min/1.7 m^2^.

The tumour markers showed CA125 was 7.4 KU/L (normal ≤ 35), CA19.9 = 19 KU/L (normal ≤ 35), and CEA = 1.2 *μ*g/L (normal ≤ 4). The abdominal/pelvic ultrasound revealed a right ovarian mass which was confirmed on CT scan.

Laparotomy was performed, and the ovarian mass together with right fallopian tube were excised. The mass weighed 9.5 kg and measured 290 × 115 × 120 mm. Sectioning of the mass showed a multilocular cystic areas containing amber coloured, thick mucoid material alternating with multiple, solid firm nodules within the fibrous stroma of the walls. 

**Figure 1:**
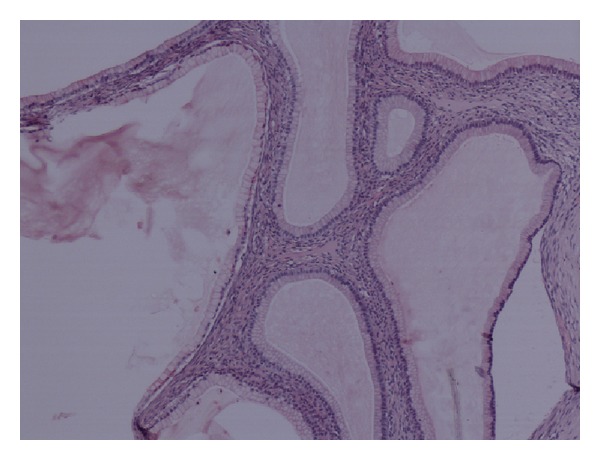
Part of the tumour showing benign mucinous cystadenoma.

**Figure 2:**
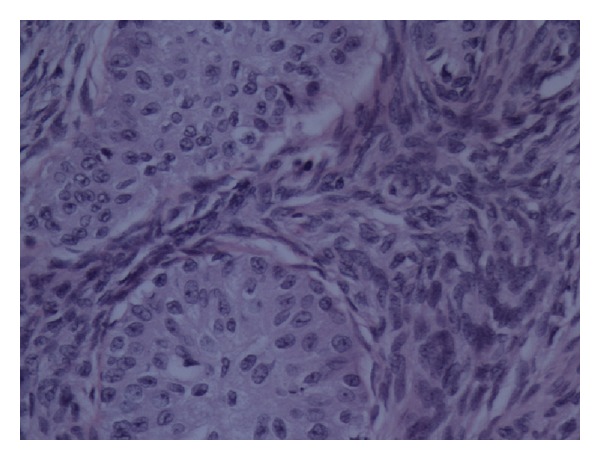
Part of the tumour showing Brenner tumour.

**Figure 3:**
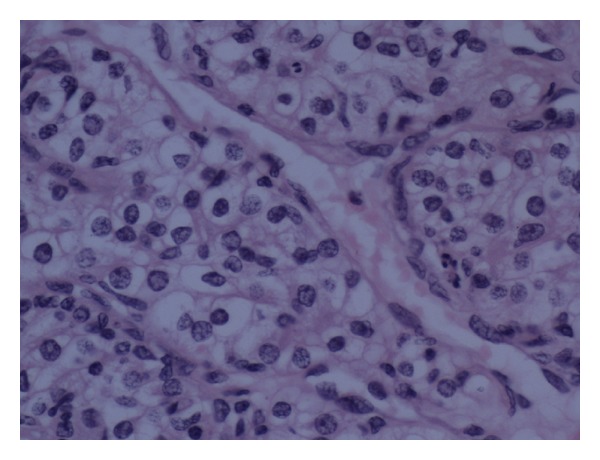
Metastatic renal cell carcinoma in the ovary, forming nests of clear cells separated by thin fibrovascular stroma.

**Figure 4:**
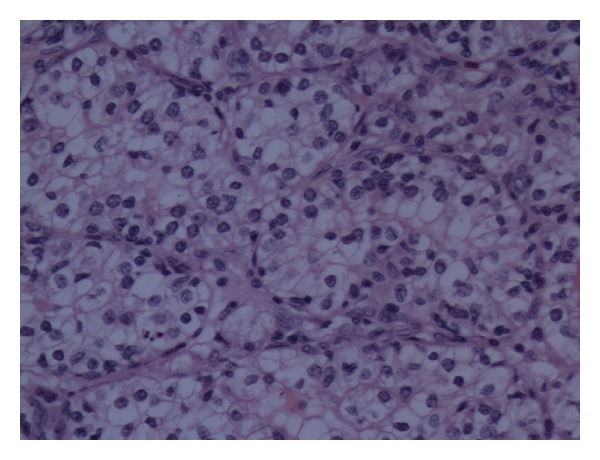
Metastatic renal cell carcinoma in the ovary.

Histological examination showed a multilocular cystic ovarian tumour which consisted of mixed benign mucinous cystadenoma and Brenner tumour (Figures 1 and 2). This mixed tumour forms the main component of the cystic mass. In addition, there were densely packed glandular structures lined by clear cells arranged in nests separated by vascular stroma. The nuclei are small, uniform with minimal pleomorphism and no prominent nucleoli or discernible mitoses (Figures 3 and 4).

The grading and nature of this tumor was difficult to assess on conventional H&E sections. The differential diagnoses were clear cell carcinoma of the ovary, carcinoid, and Sertoli cell tumor.

**Figure 5:**
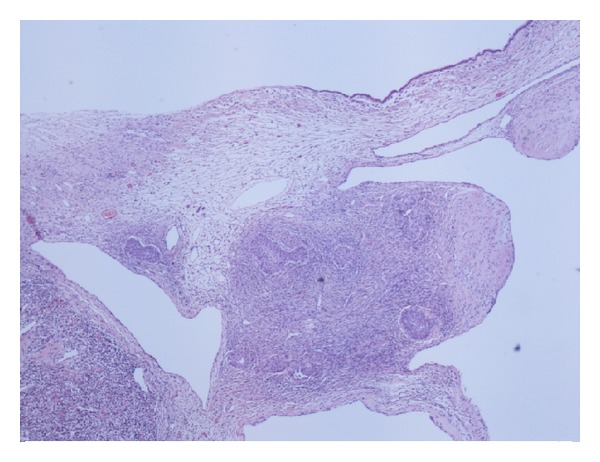
The three components: RCC, Brenner tumour, and Mucinous cystadenoma.

**Figure 6:**
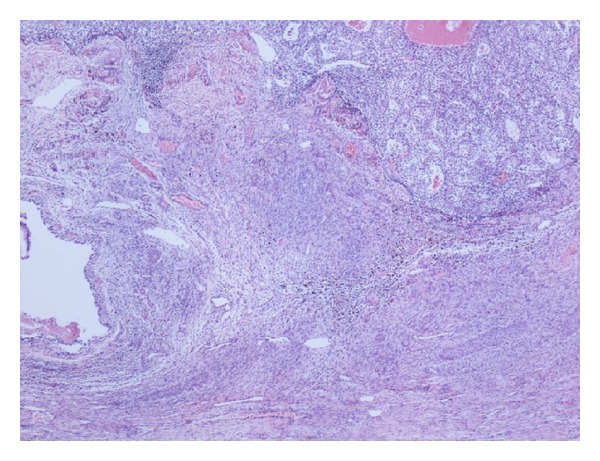
The three components: RCC, Brenner tumour, and Mucinous cystadenoma.

The clear cells were positive for immunostains CD10, Pan CK, Cam5.2, Vimentin, and EMA. The cells were negative for CK7, CK20, GP200, ER/PR, and S100. The histology and the immunostaining profile were suggestive of a metastatic renal cell carcinoma—clear cell type which is present in a benign mixed mucinous cystadenoma and Brenner tumour (Figures 5 and 6).

 Further investigation of the patient revealed the presence of a tumour in the right kidney which was confirmed as renal cell carcinoma.

## 3. Discussion

Renal cell carcinoma (RCC) is the most common malignant tumor of the kidney. It constitutes 85% of renal neoplasms in adults and 3% of all adult malignancies [1]. It usually affects people between 50–70 years of age [1]. It is more common in males; male to female ratio is 2 : 1 [4]. Approximately, 30,000 new cases are diagnosed each year, and 12,000 deaths occur due to the disease [5]. About 30% of patients have distant metastasis at the time of diagnosis [1, 4]. It usually metastasizes to other organs via lymphatic and venous routes [6]. The usual sites for metastasis of this tumour are the lungs (50–60%), lymph nodes (36%), bones (30–40%), liver (30–40%), and brain (5%) [2]. RCC is known to metastasize to other rare sites and have unpredictable biological behavior. Although ovarian metastasis is rare, sometimes it can also be the initial manifestation of the disease. The majority of ovarian tumors are primary. The most common tumors of the ovary are the surface epithelial tumors. One of the common surface epithelial tumors of the ovary is benign mucinous cystadenoma. It is usually unilateral, and affects women between the 3rd and the 5th decades of life [3]. 

Sometimes, mucinous cystadenomas are associated with other types of primary ovarian tumours such as Brenner tumour. The latter constitutes about only 2% of all ovarian tumours and has been reported in 20% of cases in association with other tumours such as benign serous, mucinous cystadenoma, or benign cystic teratoma [7, 8]. 

Secondary tumours of the ovary constitute only around 6% of ovarian tumors. The usual cancers that metastasize to the ovaries are those arising from stomach, colon, breast, and lymphomas [9]. 

Renal cell carcinomas rarely metastasize to the ovaries because these tumors usually occur during the 6th and 7th decades of life, where sclerosis of the ovarian vessels had usually occurred. Therefore, due to its rarity, clear cell carcinoma of the kidney when metastasizes to the ovaries is usually misdiagnosed as primary clear cell carcinoma of the ovary [4]. 

Sometimes, it can be difficult to differentiate between renal cell carcinoma—clear cell type and clear cell carcinoma of the ovary. However, the presence of certain histological criteria can help pathologists to differentiate between the two tumors. Tubulocystic pattern with the presence of “Hobnail cells” and extracellular mucin are more in favour of clear cell carcinoma of the ovary, whereas the presence of solid sheets of clear cells and thin wall blood vessels surrounded by a pseudocapsule formed by the compressed parenchyma and fibrous tissue are all in favour of renal cell carcinoma—clear cell type [10]. 

The differential diagnosis of primary clear cell tumour of the ovary includes primary clear cell carcinoma, Sertoli cell tumour, dysgerminoma, Lipoid cell tumour, Hilus cell and Leydig cell tumours [11]. Despite its rarity, metastatic renal cell carcinoma should also be considered in the differential diagnosis of clear cell tumor in the ovary. Immunohistochemistry has a very important role in the diagnosis. Renal cell carcinoma cells usually exhibit positive expression of PAX2, EMA, CD10, Cam5.2, and AE1/3, whereas ovarian tumor cells exhibit positive expression for CK7, CA125, and ER/PR. 

Our case highlights an unusual presentation of renal cell carcinoma that has metastasized to the ovary which was extensively involved by a mucinous cystadenoma associated with Brenner tumour.

In conclusion, a thorough clinical history, with careful evaluation of gross and microscopic features of the clear cell ovarian tumour, and use of immunohistochemical stains are extremely important in the diagnosis of rare metastatic renal cell carcinoma to the ovary. It is also important to consider the possibility of renal cell carcinoma in the differential diagnosis of any suspicious abdominal mass, in view of the unpredictable biological behaviour of this tumour [12].
